# Spotlight on the Roles of Whitefly Effectors in Insect–Plant Interactions

**DOI:** 10.3389/fpls.2021.661141

**Published:** 2021-07-02

**Authors:** Diana Naalden, Paula J. M. van Kleeff, Sarmina Dangol, Marieke Mastop, Rebecca Corkill, Saskia A. Hogenhout, Merijn R. Kant, Robert C. Schuurink

**Affiliations:** ^1^Department of Evolutionary and Population Biology, Institute for Biodiversity and Ecosystem Dynamics, University of Amsterdam, Amsterdam, Netherlands; ^2^Green Life Sciences Research Cluster, Swammerdam Institute for Life Sciences, University of Amsterdam, Amsterdam, Netherlands; ^3^Department of Crop Genetics, John Innes Centre, Norwich Research Park, Norwich, United Kingdom

**Keywords:** phloem feeders, effector proteins, insect pests, host plants, plant defense, whitefly (*Bemisia tabaci*)

## Abstract

The *Bemisia tabaci* species complex (whitefly) causes enormous agricultural losses. These phloem-feeding insects induce feeding damage and transmit a wide range of dangerous plant viruses. Whiteflies colonize a broad range of plant species that appear to be poorly defended against these insects. Substantial research has begun to unravel how phloem feeders modulate plant processes, such as defense pathways, and the central roles of effector proteins, which are deposited into the plant along with the saliva during feeding. Here, we review the current literature on whitefly effectors in light of what is known about the effectors of phloem-feeding insects in general. Further analysis of these effectors may improve our understanding of how these insects establish compatible interactions with plants, whereas the subsequent identification of plant defense processes could lead to improved crop resistance to insects. We focus on the core concepts that define the effectors of phloem-feeding insects, such as the criteria used to identify candidate effectors in sequence-mining pipelines and screens used to analyze the potential roles of these effectors and their targets *in planta*. We discuss aspects of whitefly effector research that require further exploration, including where effectors localize when injected into plant tissues, whether the effectors target plant processes beyond defense pathways, and the properties of effectors in other insect excretions such as honeydew. Finally, we provide an overview of open issues and how they might be addressed.

## Introduction

### *Bemisia tabaci* Poses a Serious Threat to Crops

*Bemisia tabaci* (Hemiptera; Aleyrodoidea) is a cryptic species complex consisting of at least 34 distinct genetic groups and 392 haplotypes (De Barro, 2012), most of which are challenging to distinguish morphologically (Boykin et al., 2013). *Bemisia tabaci* have been identified in most countries and on all continents except Antarctica (Kanakala and Ghanim, 2019; Sani et al., 2020). The Mediterranean (MED, formerly known as the Q biotype) and the Middle-East-Asia Minor 1 (MEAM1, formerly known as the B biotype or *Bemisia argentifolii*) are thought to be among the most widespread and invasive *B. tabaci* species (Boykin et al., 2013). *Bemisia tabaci* is a phloem-feeding polyphagous insect and feeding damage induced by these insects can cause crop losses with disastrous consequences for farmers, particularly smallholder farmers in developing countries. Infested plants show reduced vigor and yield due to the withdrawal of nutrients from the phloem. In addition, the sugary excretions of whiteflies (known as honeydew) form dense layers on the leaf surfaces that attract sooty molds and reduce photosynthesis (Inbar and Gerling, 2008). Honeydew on the other hand can attract natural enemies and parasitoids of whiteflies, which benefits the host plant (reviewed by Inbar and Gerling, 2008). *Bemisia tabaci* causes different types of feeding damage at the adult or nymphal stage. For example, modest infestations of adults and subsequent nymphal development can cause irregular ripening of tomato fruit (Schuster et al., 1990; Hanif-Khan et al., 1998). In cucurbits, feeding by MEAM1 causes the formation of silvery lesions on newly emerged leaves (Jiménez et al., 1995). The formation of these lesions involves the separation of the upper epidermis from the lower cell layer (Jiménez et al., 1995; Powell and Stoffella, 1995; Inbar and Gerling, 2008). Nymph colonization often induces chlorosis in young leaves of plants such as cotton (Pollard, 1955) and tomato due to decreased chlorophyll content (Buntin et al., 1993). In addition, *B. tabaci* transmits more than 200 plant viruses (Sani et al., 2020) from the following groups: Begomoviruses (e.g., tomato yellow leaf curl virus, TYLCV); Carlaviruses (Cowpea mild mottle virus, CPMMV); Criniviruses (Tomato chlorosis virus, ToCV); Ipomoviruses (Cucumber vein yellowing virus, CVYV); and Torradoviruses (Tomato torrado virus, ToTV) (Navas-Castillo et al., 2011). These viruses can cause up to 100% yield losses in crops (Brown and Bird, 1992). Plant viruses can promote the fecundity of *B. tabaci*, thereby increasing the chance that viral infections spread even further (McKenzie, 2002; Maluta et al., 2014).

### *Bemisia tabaci* Induces Plant Defense Pathways

*Bemisia tabaci* feeds from the phloem via stylet bundle penetrations. The relatively limited cellular damage caused by these insects (compared to insects such as the chewing herbivores caterpillars and beetles) may reduce detection and defense induction by the plant host, thereby facilitating infestation. Upon feeding, a complex interaction between *B. tabaci* and the host plant occurs involving saliva and both electrical and hydraulic signals (Walling, 2000). The salivary components enable *B. tabaci* to modulate host defense mechanisms, thereby increasing plant susceptibility and enhancing whitefly performance (Kempema et al., 2007; Zarate et al., 2007; Walling, 2008). Nonetheless, plants respond to whiteflies by inducing several phytohormone-mediated defense pathways. *Bemisia tabaci* MEAM1 nymphs induce the salicylic acid (SA)-dependent pathway and either suppress or do not measurably affect the expression of jasmonic acid (JA) and ethylene (ET)-regulated genes in *Arabidopsis thaliana* (Zarate et al., 2007). Adult MED whiteflies increase SA levels, while reducing JA levels in tomato leaves (Shi et al., 2014). These findings indicate that different developmental stages of both MEAM1 and MED are able to repress JA-mediated defense responses by inducing SA-mediated defense responses.

### Plant Defense Pathways Induced by Cell-Surface Receptors and Intracellular Receptors

The plant defense response comprises a network of integrated processes (Li et al., 2020). In summary, plants recognize some pathogens via surface-exposed receptors, such as receptor-like kinases (RLKs) and receptor-like proteins (RLPs). In general, plants recognize conserved pathogen components known as microbe-, pathogen-, or herbivore-associated molecular patterns (MAMPs/PAMPs/HAMPs). In addition, plants detect damage-associated molecular patterns (DAMPs) that are released from plant cells upon damage or wounding (Jones and Dangl, 2006; Steinbrenner et al., 2020). Receptor-like kinases or receptor-like proteins often require co-receptors, such as BRASSINOSTEROID INSENSITIVE 1-associated receptor kinase 1 (BAK1) and SUPPRESSOR OF BIR 1 (SOBIR1), to transduce the recognition of a HAMP/DAMP into downstream defense signaling, such as the activation of kinases and an elevated plant defense response to invaders (Liebrand et al., 2014). This process is often referred to as PAMP-triggered immunity (PTI) (Jones and Dangl, 2006). Receptor-like kinases also play a role in plant defense against phloem-feeding insects. For example, the co-receptor BAK1 mediates plant resistance to aphids (Chaudhary et al., 2014; Prince et al., 2014), and plasma membrane-localized lectin receptor kinases (OsLecRK1-OsLecRK3) enhance resistance to the rice brown planthopper *Nilaparvata lugens* and the white-backed planthopper *Sogatella furcifera* (Liu et al., 2015). Although cell surface receptors that enhance resistance to insects have been identified, whitefly-derived HAMPs that are recognized by these receptors have not yet been identified.

Plants contain resistance (*R*) genes that produce nucleotide-binding site leucine-rich repeat (NBS-LRR) proteins that recognize pathogen effectors or their activities intracellularly. *R* genes can be further divided into the CC-domain-containing and TIR-domain-containing subfamilies (McHale et al., 2006). Recognition often leads to a hypersensitive response (HR) and immediate cell death, a process referred to as effector-triggered immunity (ETI) (Jones and Dangl, 2006; Dangl et al., 2013). All *R* genes that provide resistance to phloem-feeding insects identified to date are CC-NBS-LRRs. These include some brown planthopper *N. lugens* resistance genes (Balachiranjeevi et al., 2019; Li et al., 2019; Yang et al., 2019; Yuexiong et al., 2020) and the aphid resistance genes *Vat* (Boissot et al., 2016), *Nr* (Van Helden et al., 1993), and *Mi-1.2* (Milligan et al., 1998). Beyond *Mi-1.2*, which provides some level of resistance to the whiteflies MEAM1 and MED (Nombela et al., 2003), *R* genes that provide resistance to whiteflies have not yet been identified. Moreover *Mi-1.2* is not functional at high temperatures (Nombela et al., 2003), which is unfortunate given that whiteflies are particularly prevalent in warmer climates. To better understand the various stages of the plant immune response, the “zigzag” model was proposed (Jones and Dangl, 2006). In this model, PTI is depicted as an elevated plant defense response (“zig”), effector-triggered susceptibility (ETS) as the pathogen-mediated suppression of PTI (“zag”), and ETI as a powerful increase in the plant defense response to counteract the pathogen (“zig”), whereas the pathogen evolves (new) effectors to overcome this defense (“zag”). While this model has been useful for unraveling the various processes that define the outcome of plant–biotic interactions, more recent data indicate that PTI and ETI are not separate processes but are mechanistically connected (Thomma et al., 2011; Pruitt et al., 2020).

### The Definition of Pathogen-Produced Effectors

Pathogens and pests that successfully colonize plants have evolved mechanisms to overcome plant PTI and ETI. In plant pathology research, the word “effector” refers to “a *molecule from a plant eater that alters host-cell structure and function”* (Hogenhout et al., 2009). This definition includes elicitors, (a) virulence factors, and PAMPs. Thus, an effector may have a positive or negative effect on a plant under attack, depending on the plant's ability to directly or indirectly detect the effector and respond in the appropriate manner. Indeed, effectors that were shown to suppress immunity and promote pathogen/pest colonization in one plant species or variety can evoke an HR or induce overall plant immunity, leading to reduced colonization, in another plant species or variety. Therefore, the classification of effectors can be highly context dependent and is often difficult based on only a few experiments. Effectors can also influence processes beyond plant immunity, such as altering plant development (MacLean et al., 2011; Sugio et al., 2011, 2015) or initiating gall formation (Korgaonkar et al., 2020). There is special interest in effectors that have evolved for the purpose of modulating host plant responses, especially host defense responses (Shiraishi et al., 1992), and in the counter-adaptations of plants to undo or bypass these modulations (Dangl, 1994). In the literature, “effectors” often refers to proteins secreted during feeding, but there are also examples of non-protein molecules that function as effectors, such as coronatine (Bender et al., 1999) and RNAs (Weiberg et al., 2013; Chen et al., 2020), and not all effectors are derived from saliva (Gouhier-Darimont et al., 2019). The ability of several effectors to modulate the host's physiology is dependent on specific host proteins referred to as susceptibility proteins or S proteins (Van Schie and Takken, 2014). S proteins are not involved in pathogen recognition but have other functions that indirectly facilitate the pathogen. Abolishing the expression of an *S* gene will therefore lead to (partial) resistance to the pathogen. Conversely, the ability of plants to recognize effectors can depend on the presence of RLK/RLP receptor proteins or R proteins (Kourelis and van der Hoorn, 2018). Therefore, the absence/presence of *S* genes and *R* genes together is the main determinant of the impact of an effector on virulence and thus the effector's “identity.” In this review, we will focus on effectors that were shown (in one or more plant–biotic interaction) to contribute to increased compatibility or are expected to do so (referred to as putative effectors). Effectors that were shown (in one or more plant–biotic interaction) to increase incompatibility are referred to as elicitors or avirulence factors.

### The Topic of This Review

In the past decade, it has become clear that herbivorous arthropods produce effector molecules that modulate plant defense responses. Most studies of phloem-feeding insects have been performed with aphids and planthoppers, but several whitefly effector proteins were recently identified as well. The identification and functional analysis of these effectors is insect independent, as are studies of their modes of action and the identification of interacting plant proteins. This review will focus on effectors identified from *B. tabaci* and put these findings into the context of what is known about effectors from other phloem-feeding insects and plant-colonizing organisms. We will also critically discuss techniques used to identify and functionally characterize effector proteins and tools to identify and confirm their interacting partners.

## The Identification of Effectors

### Effector Factories: The Glands

Effector proteins are often secreted by the salivary glands of phloem feeders. Whiteflies and other hemipterans contain two types of salivary glands: the principal or primary salivary glands and the accessory salivary glands (Ponsen, 1972; Wayadande et al., 1997; Ghanim et al., 2001; Su et al., 2012; Ammar et al., 2017). In *B. tabaci*, the primary salivary glands are located in the prothorax near the head and consist of at least 13 symmetrical cells. The accessory glands are located near the anterior part of the prothorax behind the primary salivary glands and consist of four cells. In both types of salivary glands, the cells contain microvilli lined into the central lumen of the gland. The gland lumens empty into primary or accessory salivary gland ducts, which are connected to each other (Ghanim et al., 2001). The primary salivary glands of all hemipterans investigated thus far contain multiple cell types that each have different kinds of electron-dense secretory vesicles (Sogawa, 1968; Wayadande et al., 1997; Ghanim et al., 2001; Reis et al., 2003; Ammar et al., 2017; Dai et al., 2019), and produce and secrete salivary components such as proteins (Sogawa, 1968; Mutti et al., 2008; Yang et al., 2017; Su et al., 2019; Xu et al., 2019; Huang et al., 2020), long non-coding RNAs (Chen et al., 2020), and small RNAs (Van Kleeff et al., 2016). Some of these secreted salivary component are effectors or elicitors, but some have other functions, such as structural roles in salivary sheaths (Cohen et al., 1998; Freeman et al., 2001; Will and Vilcinskas, 2015), and others may play a role in both (Shangguan et al., 2018).

The salivary transcriptome varies with different diets or plant species (Jonckheere et al., 2016; Rivera-Vega et al., 2018: Huang et al., 2020), the presence of viruses (He et al., 2020) or endosymbionts (Wang et al., 2020). For example, TYLCV alters gene expression in *B. tabaci* salivary glands where it replicates and this also occurs in the presence of the non-replicating papaya leaf curl China virus (PaLCuCNV) (He et al., 2020). In *B. tabaci*, the endosymbiont *Rickettsia* alters the transcriptome of whiteflies that colonize cotton (Kliot et al., 2019) and we speculate that these alterations might also occur in salivary glands. In other phloem feeders, symbionts can also induce the transcription of putative effector genes. For example, the aphid *histidine-rich Ca*^2+^*-binding protein-like* (*ApHRC*) gene is upregulated in salivary glands when the secondary symbiont *Serratia symbiotica* is present (Wang et al., 2020). Although ApHRC has effector properties it has not yet been shown to be secreted. Changes in the transcriptome most likely also affect the proteome of *B. tabaci* saliva, and the effector proteins therein, as was shown for the generalist spider mite *Tetranychus urticae* whose salivary transcriptome and proteome is strongly dependent on host plant identity (Jonckheere et al., 2016, 2018). In summary, the salivary glands produce effectors, and the expression of corresponding genes can vary depending on the plant host, and the presence of (endo)symbionts or plant viruses.

### Effectors From Other Sources

Although the majority of effectors are secreted from salivary glands, effectors may come from other sources as well, including from other organisms. For example, effectors of bacterial plant pathogens such as phytoplasmas promote plant colonization of their insect vectors like leafhoppers, planthoppers, and psyllids (reviewed in Tomkins et al., 2018; Huang et al., 2021). *Bemisia tabaci* depends on endosymbionts to produce essential amino acids that phloem lacks. These symbionts include the primary (obligate) bacterial symbiont *Portiera aleyrodidarum* and one or more secondary (facultative) bacterial symbionts such as *Hamiltonella, Wolbachia*, and *Rickettsia* species. *Portiera* is vertically transmitted via the female line into the developing egg before it is laid, while the secondary symbionts may be both vertically and horizontally transmitted (Skaljac et al., 2017). The presence of the secondary symbionts in whiteflies is geographically specific and affects whitefly fitness, reproduction, host plant defense, insecticide susceptibility, adaptation to stress, thermal tolerance, or viral transmission (Gottlieb et al., 2010; Brumin et al., 2011; Himler et al., 2011; Rana et al., 2012; Civolani et al., 2014; Su et al., 2014, 2015; Rao et al., 2015; Ghosh et al., 2018; Kanakala and Ghanim, 2019). Saliva of the aphid *Macrosiphum euphorbiae* contains proteins that originate from the primary endosymbiont *Buchnera aphidicola* (Chaudhary et al., 2014). One of these proteins is GroEL, a heat-shock protein (chaperone), that induces PTI in *A. thaliana* (Chaudhary et al., 2014). GroEL has also been identified in *B. tabaci*, where it is produced by the insect's secondary endosymbiont *Hamiltonella* (Gottlieb et al., 2010). Carrying *Hamiltonella defensa* promotes whitefly–plant interactions by suppressing JA and JA-induced anti-herbivore defense responses (Su et al., 2015).

Honeydew is secreted by whiteflies and accumulates around the feeding site and on the leaves below, where it induces plant immune responses. Applying honeydew from whiteflies or aphids increases endogenous SA accumulation in the plant (Schwartzberg and Tumlinson, 2014; VanDoorn et al., 2015). Although more than 80% of the SA present in honeydew is converted into the inactive glycoside form (SAG), it still appears to be able to induce endogenous SA accumulation (VanDoorn et al., 2015). The honeydew of whiteflies likely also contains proteins. For example, the honeydew of the pea aphid *Acyrthosiphon pisum* contains not only proteins from the insect itself, but also from endosymbionts, such as GroEL (Sabri et al., 2013). The honeydew of the planthopper *N. lugens* was recently found to induce plant defense responses via its honeydew-associated microbiota. These microbiota induce the production of volatile organic compounds (VOCs) and phytoalexins in both rice cells and seedlings and activate diterpene-based defense responses (Wari et al., 2019a,b).

The detection of herbivore eggs by the plant induces defense responses, as their presence poses an important threat to the plant (Reymond, 2013). In *A. thaliana*, the lectin receptor kinase LecRK-I.8 is involved in the perception of *Pieris brassicae* eggs (Gouhier-Darimont et al., 2019). Recently, it was shown that *A. thaliana* induce plant defenses to an egg-associated glandular secretion of *P. brassicae* (Paniagua Voirol et al., 2020). Furthermore, phosphatidylcholines are released from *P. brassicae* eggs, resulting in SA and H_2_O_2_ accumulation, the induction of defense gene expression, and cell death in *A. thaliana* (Stahl et al., 2020). In addition, secretions from the oviduct of *Diprion pini* function as an elicitor of the systemic release of pine volatiles to attract the insect's enemy (Hilker, 2005). Whitefly eggs are secured to the leaf by the pedicel which is a hook-like structure, which extends beyond the egg chorion, and this structure is inserted directly into a slit created in the epidermal cells by the female ovipositor and is surrounded by a glue-like substance called cement (Paulson and Beardsley, 1985; Buckner et al., 2002). The pedicel functions in the uptake of water from the plant tissue to maintain the proper balance of water in the egg (Gameel, 1974). In addition, *B. tabaci* eggs are able to take up water-soluble, membrane permeable compounds via the pedicel (Buckner et al., 2002). It remains unclear whether eggs actively secrete effectors into plant tissue, as postulated in Reymond (2013). It is clear that one effector of *B. tabaci* is higher expressed in eggs compared to all nymphal stages (Yang et al., 2017), although its function in the egg remains to be determined.

### Pipeline for the Identification of Effectors

The majority of whitefly effector proteins that have been described to date were detected by identifying transcripts encoding proteins with signal peptides that lack transmembrane domains (beyond the signal peptide) in transcriptome data (Su et al., 2019; Wang et al., 2019; Xu et al., 2019). This type of transcriptome data mining is commonly used to identify effectors from insect herbivores, including phloem-feeding insects (Bos et al., 2010; Hogenhout and Bos, 2011; Zhang et al., 2017; Pacheco et al., 2020). This mining tool is relatively easy to use and has led to the identification of many putative effectors. Other uses for transcriptome data in search for putative effector genes is determining gene expression under different environmental circumstances which could alter the expression of effector genes (Jonckheere et al., 2016; Malka et al., 2018; Rivera-Vega et al., 2018; Wang et al., 2019, 2020; He et al., 2020; Huang et al., 2020). Also, the analysis of the transcriptomes of different *B. tabaci* species on different host plants could point to effector genes that are specifically induced, as was shown for the aphid species *Myzus persicae* (Mathers et al., 2017; Chen et al., 2020), *Myzus cerasi* (Thorpe et al., 2020), and *A. pisum* (Eyres et al., 2016; Boulain et al., 2019).

Transcriptome analysis can generate a long list of putative effectors; thus, a well-thought-out selection process is required. Selection can be based on high similarity with other known effectors in other insects. Conversely, proteins essential for processes such as the regulation of gland cells can be excluded from selection. However, most proteins might have unknown functions and therefore, even selection based on the presence of signal peptides, the absence of transmembrane domains, and specific expression in salivary glands or on a particular host can generate a long list of putative effectors. Most bioinformatics data mining strategies in the field of phloem feeders is based on an aphid study (Bos et al., 2010). The presence of amino acid polymorphisms in putative effectors in two aphid species is used as a selection criterion in this pipeline and these polymorphisms are confirmed to be important for effector activities (Pitino and Hogenhout, 2013; Rodriguez et al., 2017; Escudero-Martinez et al., 2020). A similar study between MEAM1 and MED to investigate whether these genes are evolving could be an important step in the identification of additional whitefly effectors in the future. When searching for genes that confer durable resistance, there may be a benefit to look for effector genes that evolve less rapidly as such effectors are more likely to have essential functions for the insects and less likely to accumulate mutations that overcome plant resistance (Drurey et al., 2019).

In addition to bioinformatics data mining, analysis of proteomics data or measuring enzymatic activity in artificial diets has been shown to predict effector proteins for whiteflies, aphids, and planthoppers (Carolan et al., 2009; Cooper et al., 2010; Yang et al., 2017; Huang et al., 2018; Su et al., 2019). In a recent study, both salivary transcriptomic and saliva proteomic data were obtained for *B. tabaci* (MED) (Huang et al., 2020). Interestingly, the overlap between the identified proteins was rather small. Of the 171 proteins identified in the saliva proteome, only 45 were predicted from the transcriptomic data. In addition, of these 171 proteins, only 50 contained a signal peptide. Therefore, it appears that transcriptomic analysis is limited because it might exclude proteins that are somehow secreted by the whitefly into plant tissues via routes other than the endoplasmic reticulum (ER)-Golgi pathway (Rabouille, 2017) or are missed due to the limitations of the RNA sequencing technique itself (Oppenheim et al., 2015). An additional limitation is that not every protein with a signal peptide is secreted by the whitefly into the plant but is instead involved in cellular processes in the whitefly. Another point discussed by Huang and co-workers is that they did not find previously published effectors of whiteflies in their data set, indicating that different environmental conditions or diets might lead to the production of different cocktails of effectors in different studies (Huang et al., 2020).

A challenging approach to identifying effectors secreted into plant tissue is to perform proteomic analysis on tissue from which the whitefly feeds. This approach might be better than transcriptome mining and proteomic analysis of artificial diets, since the proteins identified by this analysis would actually be injected into the plant tissue. However, this would also lead to the identification of many plant proteins, and the concentrations of effectors might be rather low. Proteomics of phloem exudates is another approach used to identify whitefly effectors, but since these effectors likely enter cells and move from cell to cell, their concentrations are bound to be very low as well. In addition, the effectiveness of detecting proteins in plant material is also dependent on the availability of well-annotated genomes for both the host and insect. Finally, as whiteflies form a whole with their microbial symbionts, the transcriptome analysis should be extended to include the (partly prokaryotic) holobiome. Plant proteins that functionally interact with such secondary effectors can be used for resistance breeding just the same since the insect's well-being is often strongly dependent on a stable interaction with their symbionts (Sugio et al., 2015).

### Identified Whitefly Effectors

**Figure 1:**
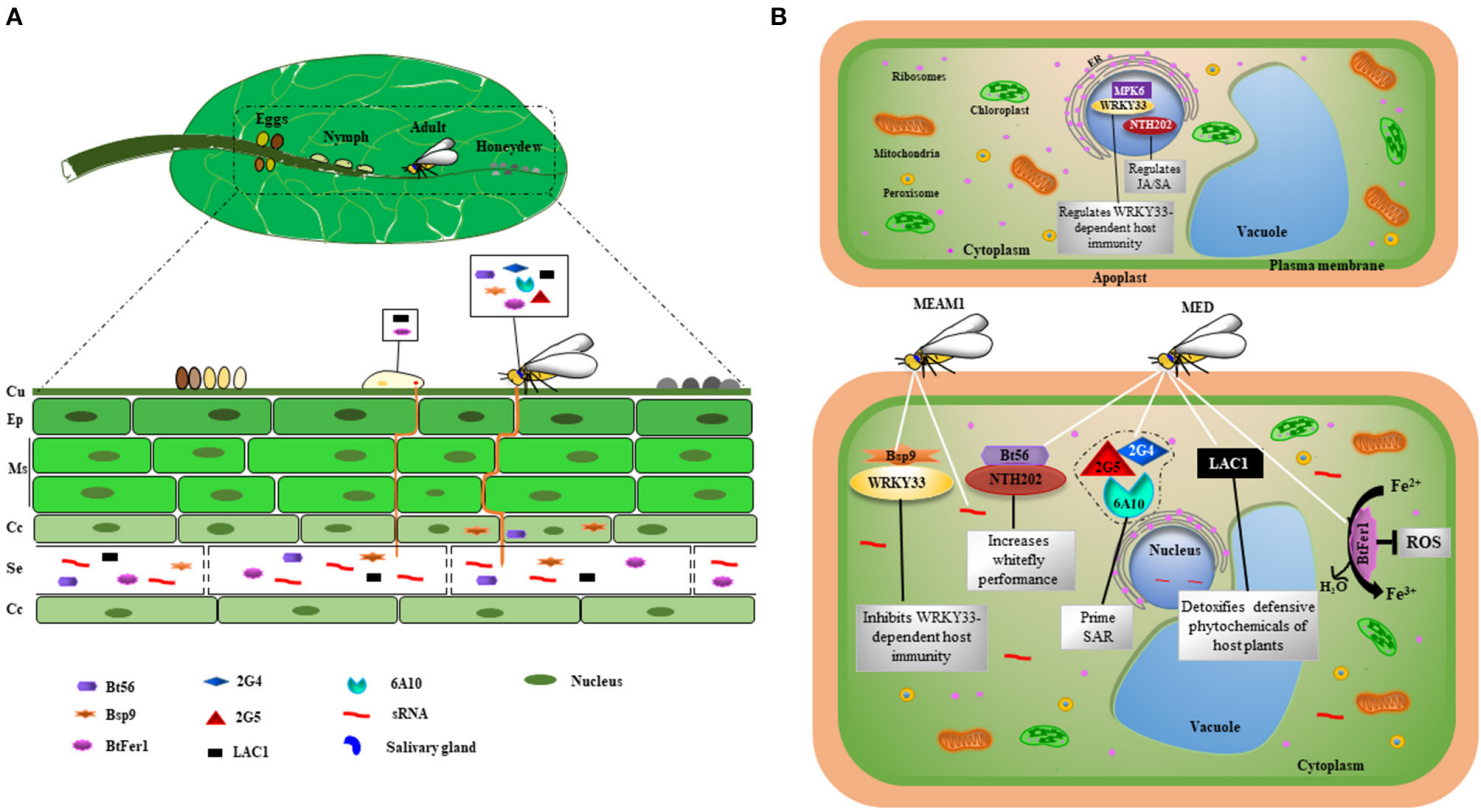
Effectors secreted during *B. tabaci* infestation in plant tissue and their proposed modes of action. **(A)** Stages of *B. tabaci* development on the abaxial surface of the leaf. Yellowish eggs darken as they mature. The stylets from *B. tabaci* nymphs and adults puncture plant tissue in order to reach phloem sieve tube elements and release watery saliva containing effectors, which interfere with plant defense responses. *Bemisia tabaci* secretes sticky, sugary honeydew on the leaf surface. *Bt56, Bsp9, 2G4, 2G5, 6A10, BtFer1*, and *LAC1* are expressed in salivary glands of adult *B. tabaci*, whereas *LAC1* and *BtFer1* are expressed in nymphs. The sRNAs and the effector BtFer1 are localized to the phloem, and Bt56, LAC1, and Bsp9 are also likely secreted into the phloem. (**B)** Modes of action of whitefly effectors in host cells. *Bemisia tabaci* MEAM1 releases Bsp9 and sRNAs into plant cells, whereas MED releases Bt56, LAC1, BtFer1, 2G4, 2G5, and 6A10. Bsp9 and Bt56 target transcription factors and keep them in the cytoplasm, inhibiting their activity in the nucleus. Bsp9 interacts with WRKY33 in the cytoplasm, thereby disrupting the interaction between WRKY33 and the pathogen-responsive MPK6 in the nucleus, resulting in increased host susceptibility. Bt56 targets tobacco KNOTTED 1-like homeobox (KNOX) NTH202 in the cytoplasm. BtFer1 convert ferrous iron to ferric iron, thereby suppressing the production of H_2_O_2_-generated oxidative signals. LAC1 helps *B. tabaci* detoxify defensive phytochemicals. 2G4, 2G5, and 6A10 induce systemic acquired resistance in the host plant upon exposure to the soil-borne pathogen *Ralstonia solanacearum*. ER, endoplasmic reticulum; SAR, systemic acquired resistance; Cu, cuticle; Ep, epidermal cells; Ms, mesophyll cells; Cc, companion cells; Se, sieve tube elements.

The presence of effectors in the saliva of phloem-feeding insects in general has been recognized for several years. De Vos and Jander showed that injection of *M. persicae* saliva into *A. thaliana* leaves caused local aphid resistance. Subsequent fractionation of *M. persicae* saliva lead to a 3–10 kDa proteinaceous fraction responsible for this resistance (De Vos and Jander, 2009). Supplementary Table 1 depicts articles that have been published on aphid, planthopper and psyllid effectors. To date, six studies describing whitefly effectors have been published (Table 1) (Van Kleeff et al., 2016; Yang et al., 2017; Lee et al., 2018; Su et al., 2019; Wang et al., 2019; Xu et al., 2019). These effectors and their *in planta* locations and modes of action are illustrated in Figures 1A,B, respectively. The first evidence that whiteflies indeed secrete molecules into plant tissue was demonstrated by a study by van Kleeff and co-workers. This study shows that sRNAs originating from *B. tabaci* are present in phloem exudates of whitefly-infested tomato plants. Although not yet confirmed, this finding suggests that these sRNAs act as effectors by interfering with gene expression in host cells (Van Kleeff et al., 2016). The silencing of host genes by exogenous sRNAs has been demonstrated for several pathogenic organisms such as the fungus *Botrytis cinerea* (Weiberg et al., 2013; Wang et al., 2017) and the parasitic plant *Cuscuta campestris* (Shahid et al., 2018).

**Table 1:** Putative effectors of *B. tabaci*.

**Putative effector name**	**Proposed mode of action**	**Additional putative effectors**	**References**
Small RNAs	Unknown	No	Van Kleeff et al., 2016
Laccase 1	Allows whiteflies to overcome the chemical defenses of the host plant	Homologs LAC, LAC2, and LAC4 not functionally analyzed	Yang et al., 2017
2G4 2G5 6A10	Reduce disease development caused by the leaf pathogen *P. syringae* pv. *tabaci* and the soil-borne pathogen *R. solanacearum*. Prime expression of SAR marker genes *NbPR1a* and *NbPR2* in local and systemic leaves in response to *P. syringae* pv. *tabaci*	No	Lee et al., 2018
BtFer1	Exhibits Fe^2+^ binding ability and ferroxidase activity, thereby suppressing H_2_O_2_-generated oxidative signals in tomato	Homologs BtFer2, BtFer3, BtFer4, and BtFer5 not functionally analyzed	Su et al., 2019
Bsp9^*^	Suppresses DAMP-induced plant immunity induced by the elicitor Pep1 by interacting with host immunity regulator WRKY33	Bsp1^(+)^, Bsp2^(0)^, Bsp3 (lectin)^(−)^, Bsp4^(0)^, Bsp5^(+)^, Bsp6^(0)^, Bsp7^(−)^, Bsp8^(−)^, and Bsp10^(0)^ screened for their ability to affect induction of DAMP-induced plant immunity on *N. benthamiana* leaf by the elicitor Pep1: no effect (0), increased *PDF1.2* activity (+), decreased LUC activity (–)	Wang et al., 2019
Bt56^*^	Activates the SA pathway and interacts with a plant KNOTTED 1-like homeobox transcription factor (NTH202)	Orthologs from Asia II 3, Asia II 1, and China 2	Xu et al., 2019

* *Bt56 and Bsp9 are orthologous effectors in MED and MEAM1, respectively; there is one amino acid difference between these two effectors according to the NCBI database*.

The first whitefly effector analyzed for its mode of action was laccase 1 (LAC1), which was identified in the salivary gland transcriptome of *B. tabaci* (MED) (Yang et al., 2017). LAC1 belongs to the blue copper-containing polyphenol oxidase family and harbors three Cu-oxidase domains typical for this family; these domains are conserved in several laccases of other insect species (Yang et al., 2017), and are thought to be important for metal ion metabolism, lignocellulose digestion, and detoxification of specialized plant metabolites (Dittmer et al., 2004; Coy et al., 2010; Yang et al., 2017). The LAC1 protein is secreted by *B. tabaci*, which was confirmed by the detection of LAC1 enzymatic activity in artificial diet. *LAC1* is expressed at all developmental stages including eggs. The highest expression of *LAC1* is seen in the adult salivary glands, but expression can also be detected in the midgut (Yang et al., 2017). The expression of *LAC1* is influenced by the host and is higher when *B. tabaci* fed on tomato plants compared to an artificial diet. Reduced expression of *LAC1* by RNA interference (RNAi) decreases the survival rate of *B. tabaci* adults feeding on tomato plants but not on artificial diet. In addition, the expression of *LAC1*, both in salivary glands and the midgut, increases when whiteflies feed on JA-treated plants compared to control plants. Taken together with the evidence of LAC1 secretion in the artificial diet, this suggests that LAC1 helps *B. tabaci* to overcome plant defense responses and may act as effector in the plant cell (Yang et al., 2017).

Several effectors were identified by screening a cDNA library of *B. tabaci* MED based on their capacity to suppress the HR caused by *Pseudomonas syringae* pv. *tabaci* or *P. syringae* pv. *syringae* (Lee et al., 2018). Of the 893 cDNAs tested, three effectors (2G4 and 2G5 encoding proteins with unknown function, and 6A10, a partial transcript of a large subunit ribosomal RNA) were selected using this bioassay. Also, transient expression of these effectors primes the expression of systemic acquired resistance (SAR) marker genes *NbPR1a* and *NbPR2* in both local and systemic leaves compared to the control. 2G4 and 6A10 also induce the expression of SAR genes in the roots of plants exposed to the soil-borne pathogen *Ralstonia solanacearum*, whereas 2G5 only induces the expression of *NbPR2*. However, all three effectors decrease the symptoms induced by *R. solanacearum*. These effectors, or effector-induced signaling molecules, might be able to translocate between cells, leading to the priming of SAR-related genes (Lee et al., 2018). However, the underlying mechanism has not been further investigated. Both *2G5* and *6A10* are expressed in the salivary glands of whiteflies when feeding on cucumber plants, and high expression in the midgut is also observed for *6A10*. The effector gene *2G4* is not expressed when whiteflies feed on cucumber (Lee et al., 2018).

The whitefly effector BtFer1 is a member of the ferritin-like superfamily with a ferritin-like domain at position ^44^Y–^202^M. BtFer1 was selected for further study for its putative mode-of-action on reactive oxygen species (ROS) production. Reactive oxygen species signaling is an important mechanism used by plants against phloem feeders and other insect herbivores (reviewed in Kerchev et al., 2012). *BtFer1* was identified in the genome of *B. tabaci* (MED) (Xie et al., 2017; Su et al., 2019). In addition, *BtFer1* shares 56–58% similarity with ferritins of other phloem feeders such as *M. persicae, A. pisum*, and *Diuraphis noxia*, but the mode of action of these proteins has not yet been analyzed. Four other ferritin genes were identified in the genome of *B. tabaci*, but these genes share only 19% similarity with *BtFer1* (Su et al., 2019). The ability of BtFer1 to bind ferrous iron and its ferroxidase activity was confirmed in this study as well. *BtFer1* is expressed equally in the salivary glands and midgut and higher during all *B. tabaci* feeding stages compared to non-feeding stages, indicating that BtFer1 plays a role during all *B. tabaci* feeding stages. Excitingly, the authors show that BtFer1 is secreted into the tomato phloem and suppresses H_2_O_2_-mediated oxidative signaling when whiteflies are feeding, confirming the hypothesis that ROS signaling is inhibited in sieve elements. Furthermore, BtFer1 suppresses other plant defense responses including callose deposition, proteinase inhibitor activation, and JA-mediated signaling pathways. Silencing *BtFer1* reduces the duration of phloem ingestion and the survival rate of females on tomato plants (Su et al., 2019).

The *B. tabaci* (MEAM1) effector Bsp9 was identified by comparing the transcriptomes of whiteflies with and without TYLCV (Wang et al., 2019). Bsp9 is secreted into tomato leaves, as Bsp9 was detected in protein extracts from infested leaves. *In planta* expression analysis revealed that this protein accumulates in the cytoplasm where it interacts with the transcription factor WRKY33; this interaction was observed as cytoplasmic speckles in bimolecular fluorescence complementation (BIFC) assays. The Bsp9–WRKY33 interaction prevents WRKY33 from localizing to the nucleus (Wang et al., 2019). WRKY33 is required for the activation of the pathogen-responsive mitogen-activated protein kinases MPK3 and MPK6, and Bsp9 interferes with the interaction between WRKY33 and MPK6 (Mao et al., 2011; Wang et al., 2019). The role of Bsp9 in modulating the immune response is confirmed in *Nicotiana benthamiana* leaves where it reduces the *PDF1.2* promoter activity induced by the DAMP immunity elicitor Pep1. The ability to suppress this DAMP immunity response is also observed for three other effectors (Bsp3, Bsp7, and Bsp8), whereas this response is actually stronger induced in the presence of the effectors Bsp1 and Bsp5. These effectors were not analyzed further in this study, but additional analysis could provide more insight into DAMP-triggered responses by the host against whiteflies. Bsp9 is highly conserved in both the MEAM1 and MED.

Bt56, an ortholog of the MEAM1 Bsp9, was selected from a published transcriptome of *B. tabaci* (MED) salivary glands (Su et al., 2012). *Bt56* is expressed in both adults and nymphs but very low in eggs. In addition, *Bt56* is highly expressed in salivary glands compared to midgut or ovaries. The secretion of this effector into plant tissue was demonstrated in *Gossypium hirsutum* (cotton) protein extracts. *In planta* expression of *Bt56* in *Nicotiana tabacum* increases the insect's survival and fecundity, while knockdown of this effector gene by RNAi in both *N. tabacum* and *G. hirsutum* decreases the performance of whitefly (Xu et al., 2019). Knockdown of *Bt56* interferes with feeding by reducing the duration of phloem ingestion. *In planta* expression of *Bt56* results in the increased production of SA but does not influence the levels of JA or JA-Ile, neither significantly changed the transcript levels of marker genes in the JA-signaling pathway. Bt56 interacts with the KNOTTED 1-like homeobox (KNOX) transcription factor NTH202 in punctate structures in tobacco cytoplasm, as visualized by BiFC. This localization suggests that, like Bsp9, Bt56 is retaining a transcription factor from moving to the nucleus, preventing its function. Some SA- and JA-pathway genes are regulated by KNOX1 in maize. However, Xu and co-workers were careful to suggest that the altered SA levels caused by Bt56 were a direct result of this interaction, since SA levels in *N. tabacum* did not significantly change when *NTH202* expression was silenced, but whitefly performance was improved (Bolduc et al., 2012; Xu et al., 2019).

It is exciting that two independent research groups identified the highly conserved orthologs *Bsp9* and *Bt56* as effector genes. They also identified two different transcription factors as their plant targets, which the interacting effectors inhibit localization of these transcription factors to the nucleus despite these proteins differing only one amino acid; Bt56 contains an asparagine at position 30, while Bsp9 contains an isoleucine at this position (Xu et al., 2019). The two effectors might even interact with both target proteins and this hypothesis is, at least partly, confirmed by the finding that the Bt56 ortholog of MEAM1 (Bsp9) indeed interacts with NTH202 in yeast; this interaction is also confirmed with *Bt56* orthologs from the Asia II 3, Asia II 1, and China 2 species (Xu et al., 2019). Interestingly, although Bt56 from Asia II interacts with NTH202, the SA levels of Asia II 3-infested did not differ significantly from MED-infested *N. tabacum* plants. These findings help confirm the hypothesis that the interaction between Bt56 and NTH202 indirectly manipulates SA levels. It is not known if Bsp9 manipulates SA levels in the host, and therefore, we can only speculate that the reduction in JA levels occurs due to the induction of SA levels.

The number of whitefly effectors identified to date is most likely the tip of the iceberg. For example, in a search for genes exclusively expressed in the salivary glands, no fewer than 295 genes were predicted to encode proteins secreted from the salivary glands that might function as effectors in plant tissue (Su et al., 2012). In addition, recent proteomic and transcriptomic analyses of *B. tabaci* identified 698 salivary gland-enriched unigenes and 171 salivary proteins, 74 of which were specifically identified in the saliva, including 34 specifically from *B. tabaci* (Huang et al., 2020). Indeed, the interaction between the host and whitefly is complicated. A complete understanding of the different modes of action of the proteins that are not involved in salivary gland structure or cellular processes is essential for providing better protection against this pest.

## Core-Effectors Between Phloem Feeders

Sap-feeding insects of the order Hemiptera have co-evolved with plants for more than 350 million years (Hogenhout and Bos, 2011). The insects share feeding behaviors by using stylet bundles to navigate and feed from plant tissues. Given this, it is not surprising that an overlapping cocktail of effectors has been identified. For example, orthologs of the Mp10 effector were identified in divergent plant-feeding but not in blood-feeding hemipterans (Drurey et al., 2019), and the *B. tabaci LAC1* effector gene is very closely related to *LAC1* found in other phloem feeders such as *Diaphorina citri, A. pisum, N. lugens*, and *Nephotettix cincticeps* (Yang et al., 2017). The *B. tabaci* effector BtFer1 shares more than 56% similarity with ferritins in *M. persicae, A. pisum, D. noxia*, and *Coptotermes formosanus* (Su et al., 2019). These effectors could be thought of as “core-effectors,” since they are present in multiple insects and potentially have similar properties. Huang and co-workers identified 171 salivary gland proteins via mass-spectrometry and found that 97 of these proteins have putative orthologs in 22 other arthropod species (Huang et al., 2020). This finding indicates that core-proteins are indeed widely conserved among insects, independently of their hosts; we speculate that some of these proteins are effector proteins. Whether these proteins fulfill similar functions is currently unknown, though all Mp10 orthologs investigated suppress plant ROS bursts to elicitors (Drurey et al., 2019). In contrast to core-effector proteins, some of the identified effectors appear to be specific to certain phloem feeders. For instance, sequences similar to the aphid SHP (structure sheath protein) and *Ya1* effectors and other members of the *Ya* long non-coding RNA family are not found in hemipteran insects beyond aphids (Will and Vilcinskas, 2015; Chen et al., 2020). Similarly, the effector proteins Bt56 and Bsp9 have only been reported in whiteflies (Huang et al., 2020).

## Research on the Mode of Action of Effectors

### *In planta* Expression of Effectors

Once putative effectors have been identified, their roles must be analyzed *in planta* in order to confirm their effector characteristics. Many techniques are available for this analysis and here we discuss a selection of the most common techniques used. Expressing effectors in the host plant is an important and efficacious strategy for determining whether a protein plays a role in insect–plant interactions. This technique provides the opportunity to study an effector protein separately from the cocktail of effectors that is normally secreted. These proteins can be expressed *in planta* via transient expression using *Agrobacterium tumefaciens* carrying a plasmid expressing the effector. This is commonly done in the model plant *N. benthamiana* (Rodriguez et al., 2014). One of the first steps in analysis is to determine whether plants expressing the effector are more susceptible to insects. For example, the transient expression of the *M. euphorbiae* effectors Me10 and Me23 in *N. benthamiana* increases aphid fecundity. Other examples include the transient expression of *M. persicae* effectors C002, PIntO1, and PIntO2, which lead to an increased insect performance (Pitino and Hogenhout, 2013). Increased *B. tabaci* performance is observed when *Bt56* is transiently expressed in *N. tabacum*, whereas transient expression of effectors *2G4, 2G5*, and *6A10* increases plant susceptibility to leaf and root pathogens (Lee et al., 2018; Xu et al., 2019). Alternatively, the *P. syringae* type three secretion system (T3SS) can be used to deliver effectors into plant cells such as tomato cells. This system was used to show that Me10 increases *M. euphorbiae* fecundity on tomato (Atamian et al., 2013). One has to choose which combination of delivery system and plant species works efficiently with the relevant insect. In addition, creating transgenic plants expressing an effector is also an option, as has been shown for the *M. persicae* effectors C002, PIntO1 (also known as Mp1), and PIntO2 (also known as Mp2) in *A. thaliana*, which all leads to increased aphid performance (Pitino and Hogenhout, 2013).

In transient and stable expression systems that drive the expression of transgenes via constitutive promoters, such as the commonly used CaMV35S promoter, the effector protein of interest is likely more abundant than the amount secreted by the insect, which might lead to artifacts. Also, these effector transgenes might result in more transcripts in epidermal and mesophyll cells than in the vasculature. These minor obstacles could be overcome creating transgenic plants harboring constructs with phloem-specific promoters (Pitino and Hogenhout, 2013; Javaid et al., 2016). Effectors can easily be fused to a fluorescent protein (FP), providing the opportunity to detect the *in planta* subcellular localization of the putative effector protein both transiently expressed and in stable transgenic plants. Fluorescence microscopy can be used to determine where the effector accumulates in the cell and if this location changes under different conditions or in the presence of another protein. Of course, the functionality of these effector-FP fusion proteins needs to be similar to that of non-tagged effectors. Fortunately, *B. tabaci* can feed on a wide variety of plants, including the model plants *N. tabacum* and *A. thaliana*, which can easily be used for *in planta* expression of effectors and bio-assays.

### Analyzing the *in planta* Secretion of Effectors

One of the key questions in insect-effector biology is where the effectors localize within plant cells following salivation and feeding. This information is crucial for understanding their modes of action: not only the cells but also the organelles to which effectors localize are important for their putative functions, for example, in suppressing PTI or ETI, or their interactions with plant proteins. The whitefly stylet bundle consists of paired mandibles and maxillae, which form the food and salivary canal, respectively. A whitefly feeds from phloem tissue using its stylet bundle, which migrates through the outer tissue layers mainly via the intercellular space, with limited contact with the surrounding cells before it enters the phloem. However, it is unclear whether the penetration of the stylet bundle through the epidermis occurs intra- or intercellularly (Freeman et al., 2001; Stafford et al., 2012). During the migration of the stylet, the number of intracellular punctures is significantly lower for whiteflies compared to aphids (reviewed in Stafford et al., 2012). Freeman et al. (2001) reported, using scanning electron microscopy, that in most cases the whitefly stylets penetrate through the cytoplasm of the epidermal cell (intracellular) to continue in the intercellular space of the mesophyll cell. However, others reported, using DC Electrical Penetration Graph (EPG) techniques or styletectomy and light microscopy, that stylets penetrate the epidermis intercellularly while few intracellular punctures occur when the stylet bundle is close to the phloem (Walker and Perring, 1994; Jiang et al., 1999; Stafford et al., 2012). Electrical Penetration Graph techniques show that intracellular punctures occur less frequently during whitefly feeding than during aphid feeding, consequentially whitefly feeding causes less wounding of the host plant (Walker and Perring, 1994; Jiang et al., 1999; Stafford et al., 2012). Nymphs are sedentary, but with each molt, the chitinous exoskeleton and parts of the stylet bundle is discarded (Freeman et al., 2001). Like other phloem feeders, whiteflies secrete a gel-like saliva into the intercellular space around the stylets and a watery saliva into the phloem. The gel-like saliva forms a salivary sheath around the stylet bundle (Freeman et al., 2001). Although the salivary sheath provides protection and inhibits recognition by the plant cell, it is likely that the plant still responds to sheath proteins. Therefore, it is expected that effectors are not only secreted into the phloem but also into the intercellular space, as observed for aphids (Mugford et al., 2016). Effectors may function in both the apoplast and cytoplasm as is seen for the effector Mg16820, secreted by the root-knot nematode Meloidogyne graminicola, acting as an immune suppressor in both cell compartments (Naalden et al., 2018). We speculate that stylet bundle migration in the apoplast and feeding from the sieve tube both requires the secretion of effectors.

The precise locations of some effectors of phloem-feeding insects have also been determined. An elegant study using “effector-specific” antibodies and electron microscopy shows that the *M. persicae* effector Mp10 was present in mesophyll cells adjacent to aphid stylet tracks (Mugford et al., 2016). Another immunolocalization study with tomato leaf sections indicates that the whitefly effector BtFer1 localizes to the phloem (Su et al., 2019). Ideally, FP-effector fusion proteins would be produced by whiteflies itself to follow effector localization in planta during feeding. However, this requires the generation of transgenic whitefly lines stably expressing an effector-FP fusion protein. Whereas, it is possible to knock-out genes in whitefly using the CRISPR technology (Heu et al., 2020), further technology development is needed to generate transgenic whiteflies that express FP-tagged effectors.

Cell-to-cell movement of effectors has been reported mostly in plant-pathogen studies. For instance, very detailed studies of *Magnaporthe oryzae* shows how the effectors of this fungus can move from cell-to-cell via plasmodesmata (Khang et al., 2010). Also, studies of a phloem-based phytoplasma revealed that phytoplasma effectors are unloaded from the phloem sieve cells and migrate to other cells, including mesophyll, confirming the cell-to-cell movement of effectors (Bai et al., 2009; MacLean et al., 2014). A recent study of the hessian fly *Mayetiola destructor*, a gall midge, show that some of its putative effectors remain within the attacked cells in resistant wheat cultivars but move to other cells in susceptible cultivars (Aljbory et al., 2020). For phloem feeders so far, the *M. persicae* effector Ya1 long non-coding RNA is the only one known to migrate away from the aphid feeding site to distal tissues, including other leaves (Chen et al., 2020). To what extend cell-to-cell movement occurs for whitefly effectors needs to be investigated.

### Effector Expression Patterns Through the Whitefly Lifecycle

Eggs of *B. tabaci* hatch after approximately 7 days into first instar nymphs, the crawler stage. Crawlers can walk for a few hours in a distance of several mm, to find an optimum feeding spot (Freeman et al., 2001; Simmons, 2002), where they go through three immobile nymphal stages until they reach adulthood. The time of development from egg to adult whitefly may take between 16 and 31 days depending on the plant host species and temperature (Powell and Bellows, 1992; Fekrat and Shishehbor, 2007; Sani et al., 2020). The crawler stage of whiteflies is the most sensitive stage of whitefly development. In the crawler stage, effector proteins would be essential to ensure the insect finds a suitable feeding site, as the stylet entering the leaf would probably cause a cascade of plant reactions that the insect needs to manipulate. To the best of our knowledge, the expression of putative effectors during the crawler stage has not yet been characterized. Analysis of the crawler transcriptome may lead to effectors essential for initiating feeding or infestation.

The immobility of nymphs means that they feed from a single site longer than adults and, therefore, may require different effectors and different adaptions around the area of the stylet. A molted nymph is known to penetrate the same leaf area that it fed on before molting (Freeman et al., 2001). Plant defense responses to the whitefly developmental stages may differ, and if so, effector repertoires may also differ among these stages. For instance, the *LAC1* is continuously expressed in the different nymphal stages but at lower levels compared to adult females or eggs (Yang et al., 2017). This indicates that LAC1 can play a role at all developmental stages and might play an additional role before hatching or as effector in the egg–plant tissue interaction. *BtFer1* is expressed during all stages, but at higher levels in nymphs and adult females and at the lowest levels in the pseudopupa (Su et al., 2019) indicating that btFER1 is specifically important during the feeding stages. Comparing transcriptome studies between the different nymphal stages may lead to insights into nymphal–plant interaction. Nymphal effectors can be studied *in planta* by expressing (either constitutive or with inducible promoters) or silencing the putative effector and perform fecundity or nymphal development assays which could give us insights into effectors needed for initial infestation or development. Finally, the transcript levels of effector genes may also differ depending on microbes present in the insects, as observed in the citrus psyllid *D. citri*; several effector genes were differentially expressed in adults and nymphs following infection with *Candidatus Liberibacter asiaticus* (Ca. Las) (Pacheco et al., 2020) and it may be the case with *B. tabaci*. Therefore, more research needs to be conducted on this area.

### RNA Interference to Silence Effector Gene Expression

RNA interference (RNAi) is a posttranscriptional gene-silencing mechanism that is triggered by the presence of double-stranded RNA (dsRNA) in the cell (Vogel et al., 2019). The specific silencing of one effector gene provides the opportunity to study the effects of reduced levels (or absence) of the effector protein while the other effector proteins are still present in the saliva and injected into the plant tissues. This provides insights into whether the putative effectors are involved in plant–insect interactions (Grover et al., 2019). The first RNAi study in whitefly salivary glands was performed by Ghanim et al. (2007), wherein micro-injection of dsRNA into adult whiteflies was performed, resulting in a 70% decrease in gene expression. This study was followed by several other successful efforts to silence genes in whitefly via micro-injection or other methods (reviewed in Grover et al., 2019). Delivering dsRNA via artificial diet turns out to be a successful and relative fast approach to silence gene expression in adult *B. tabaci*, including effector genes (Yang et al., 2017; Su et al., 2019; Xu et al., 2019; Xia et al., 2021).

Although it is possible to rear nymphs on artificial diet (Davidson et al., 2000), a plant-based dsRNA delivery system is a good method for investigating nymph development over time. Stable dsRNA transgenic plants has been used to silence the aphid effector genes *MpC002* and *MpPIntO2* up to 70% (Coleman et al., 2015). Silencing of the *B. tabaci v-ATPase* gene using stable transgenic lettuce results in fewer eggs due to high adult mortality and a delay in nymphal development (Ibrahim et al., 2017). In addition to stable transgenic lines, transient expression of dsRNA can be used to silence insect effector genes as shown for the *MpC002* effector (Pitino et al., 2011). Transient expression of dsRNA targeting acetylcholinesterase (AChE) or ecdysone receptor (EcR) in tobacco leaves results in a significant difference in mortality of *B. tabaci*, indicating that this method provides enough dsRNA to the phloem sieve tubes to accomplish the silencing effect (Malik et al., 2016). Similar effects are observed by using the virus-induced gene silencing (VIGS) technique in tomato to silence the *BtPMaT1* gene (Xia et al., 2021). Next to transient expression, dsRNA can be taken up by cut tomato leaflets and was successfully used to silence ecdysone pathway genes resulting in delayed development and reduced survival of whitefly during the nymphal stages (Luan et al., 2013). In summary, dsRNA, delivered in various ways, can be effectively used to silence effector genes in *B. tabaci*.

CRISPR-Cas9-based genome editing is a relatively new technique in which genes are specifically modified by the Cas9 protein complexed with a guide RNA to target DNA (Taning et al., 2017). A method was recently developed to apply this tool to adult female whiteflies called “Receptor-Mediated Ovary Transduction of Cargo,” which targets the ovary instead of using micro-injection in eggs (Heu et al., 2020). This method provides exciting options for targeting effector genes over multiple generations, which could provide insights into the function of the effector at each developmental stage.

### Immune Suppression Assays

A good immune response against insect infestation is essential for plant survival and is therefore an important target for insects. Most effector research has focused on the impacts of effectors on plant phenotypes or changes in insect performance as a first read out for immune suppression. Bos et al., 2010 pioneered the transient expression of putative hemipteran effectors in *N. benthamiana* by screening 48 putative effectors from *M. persicae*. They selected proteins for effector function based on reduced aphid fecundity (Mp10 and Mp42) or chlorosis (Mp10) (Bos et al., 2010). A similar experiment was performed for Bt56, which, when transiently expressed in tobacco, increases whitefly fecundity (Xu et al., 2019). Also for other phloem feeders transient expression studies with effectors have been done. For instance, transient expression of the *N. lugens* elicitor NlMLP in rice protoplasts decreases the viability of the plant cells. Furthermore, NlMLP expression triggers defense responses such as Ca^2+^ mobilization, the activation of MAPK cascades, and JA signal transduction, thereby reducing the performance of *N. lugens* in rice plants (Shangguan et al., 2018).

In addition to fecundity bioassays, studying the host immune response to pathogen-derived elicitors flg22 and elf8 (Zipfel, 2014) together with the effector could provide insight in any effects on PTI. It is relatively easy to measure ROS and Ca^2+^ levels, which are usually connected to the PTI response of the plant. The whitefly homolog of *M. persicae* Mp10 (Bt10) suppresses ROS production and Ca^2+^ response induced by the bacterial elicitor flg22 (Drurey et al., 2019), which induces PTI in a BAK1-dependent manner (Heese et al., 2007). Whitefly infestation in *A. thaliana* induces the expression the membrane receptor gene *PEPR1*, which also requires BAK1 for signaling (Postel et al., 2010; Wang et al., 2019) and the plant defense JA-related marker gene *AtPDF1.2*. This response can be mimicked by applying the DAMP immunity elicitor Pep1 to *A. thaliana* plants (Wang et al., 2019). This readout was also used in *N. benthamiana* to demonstrate that the whitefly effectors Bsp1 and Bsp5 increase DAMP-induced immunity, whereas four other proteins (such as Bsp9) suppress this response (Wang et al., 2019). Some insects secrete effectors to directly counteract ROS production. The proteomic analysis of salivary secretions of Cabbage looper (*Trichoplusia ni*) identified a catalase that functions as an ROS scavenger to inhibit ROS burst (Rivera-Vega et al., 2018). Similarly, the whitefly salivary protein BtFer1, secreted into plant tissue during feeding, suppresses H_2_O_2_-mediated oxidative signals in tomato (Su et al., 2019).

An additional approach to identifying the roles of effectors in plant defense is to analyze hormonal differences. Effectors can alter the expression of phytohormone-related marker genes, and effector genes can be upregulated when an insect feeds on plants treated with phytohormones. The whitefly effector Bt56 increases the expression of the SA marker gene encoding pathogenesis-related protein 1a (PR-1a) in *N. tabacum* locally following infiltration with agrobacterium. Whitefly effectors 2G4, 2G5, and 6A10 increase the expression of NbPR-1a both locally and systemically. No such phytohormone-related experiments were performed for LAC1, but the authors showed that *LAC1* expression increases when MED whiteflies feed on tomato plants sprayed with JA, compared to whiteflies that feed from control plants. The increase in *LAC*1 expression might be an indication that LAC1 is involved neutralizing the plant defense mechanism. Knocking-down *BtFer1* increases the expression of JA marker genes encoding allene oxide synthase (AOS) and threonine deaminase 2 (TD2) but not lipoxygenase D (LoxD). Taken together, these findings indicate that the whitefly effectors identified to date play various roles in manipulating hormonal pathways. We expect that in the near future, many more whitefly effectors involved in suppressing the immune response will be identified, and their exact roles and the underlying mechanisms will be further uncovered.

### Target Proteins in Plants

Identification of a target protein in the host plant provides a possible insight into the mode of action of an effector. Several techniques are available to find a target protein in the host or to confirm these interactions *in planta*. The yeast-two hybrid (Y2H) system is a relatively easy tool to identify possible host target proteins for an effector. Several target proteins of phloem-feeding insect effectors have been identified using this method (Hu et al., 2017; Rodriguez et al., 2017; Chaudhary et al., 2019; Wang et al., 2019; Xu et al., 2019). Yeast-two hybrid screens revealed that Bt56 from MEAM1, AsiaII 1, AsiaII 3, and China 2 interact with the tobacco transcription factor NTH202 (Xu et al., 2019), whereas its MED1 ortholog Bsp9 interacts with AtWRKY33. A disadvantage of Y2H is that the effector and host proteins are forced together into the nucleus of the yeast cells. Instead, in plant cells the two proteins might be in different subcellular compartments. Contrarily, interactions that occur *in planta* might not be detected in yeast because the protein was not expressed in the library used. For example, the expression of genes in the host could change in the presence of the insect (Van de Ven et al., 2000; Kempema et al., 2007; Zarate et al., 2007; Puthoff et al., 2010; Li et al., 2016), making the choice for the cDNA-Y2H library very important. A possible method to identify *in planta* interactions is affinity purification coupled to mass spectrometry (MS). For this, plant tissue expressing a tagged effector of interest is used to pull out its plant target proteins that are subsequently analyzed by MS. This method also has several disadvantages. For example, a weak interaction could be disrupted during the washing steps, or rupture of the cells during protein extraction could allow proteins that are normally located in different cellular compartments to come into contact with one another including the effector (Bontinck et al., 2018). However, the big advance is that protein extractions can be made of whitefly-infested tissue, leading quickly to biologically relevant targets, for example when certain genes are only expressed in the presence of the herbivore.

Once an effector target protein has been identified, these interactions should be further confirmed. Commonly used techniques are: (i) BiFC, where the effector and target proteins are fused to complementary halves of a yellow fluorescent protein (YFP), producing a YFP-fluorescent signal upon interaction; (ii) Förster resonance energy transfer by fluorescence lifetime imaging (FRET-FLIM), whereby energy transfer taking place between a donor and an acceptor chromophore when the two fused proteins interact is detected by fluorescence microscopy; (iii) Co-Immunoprecipitation (Co-IP), where the effector and host protein are expressed with different tags and the pull-down of one of these proteins results in the pull-down of the interacting protein as well, detectable with immunoblot analysis. One advantage of BiFC over Co-IP is that it is relatively easy to perform and weak interactions are also visible using BiFC. In addition, BiFC and FRET-FLIM reveals where the interaction takes place within the plant cell as was shown for the orthologs Bsp9 and Bt56 with WRKY and NTH202, respectively, which both occur in the cytoplasm (Wang et al., 2019; Xu et al., 2019). Co-IP is generally considered to be more reliable for confirming interactions, since these interactions are pulled out of the protein solution, which may lead to fewer false signals. However, it is still necessary to confirm that these proteins are present in the same cellular compartment. These types of assays are usually performed in model plants such as *N. tabacum* and *N. benthamiana*, even when the host protein is identified from crop libraries. Therefore, it would be interesting to perform these assays in crops as well. The identification of target proteins may lead to the identification of resistance or susceptibility genes, providing interesting targets for resistance breeding (Van Schie and Takken, 2014).

### Other Functions of Effectors

Most effector research is focused on manipulating the immune responses of plants, partly because assays based on plant immunity are relatively easy to perform. To the best of our knowledge, whitefly effectors affect plant resistance. Up to date no other functions as food digestion (Eichenseer et al., 2010), manipulating the plant's source-sink relationships (Walters and McRoberts, 2006), altering the plant's cell cycle (Goverse et al., 2000; Davis et al., 2011), gall formation (Zhao et al., 2015), or increased cell size have be linked to whitefly infestation or it's effectors. Also, not much is known about what occurs at the feeding site of whiteflies. Although less visible, specific, small changes in cell structure or cytoplasmic densities might occur in plant structures such as the phloem-associated companion cells. Ca^2+^-binding proteins in the watery saliva of the aphid *Megoura viciae* play a role in suppressing sieve-tube occlusion at sieve plates of *Vicia faba*. This has been observed for other aphid species as well (Will et al., 2009). These types of proteins are likely secreted by whiteflies as well, since unobstructed phloem is necessary to provide enough nutrients for the whitefly, especially during the immobile nymphal stages when the feeding process takes a long time. Some salivary effector proteins might also function as cofactors in taste perception by recruiting and delivering sapid molecules; these molecules, such as human tastant-binding proteins, interact with saliva and bind to receptors of taste-sensing cells (Fábián et al., 2015). In whitefly, chemosensing or tasting is thought to occur in the precibarial sensilla (Hunter et al., 1996). Finally, the gel saliva and stylet sheaths of aphids and possibily whiteflies (Will et al., 2012) contain effector proteins that function in immune suppression in the intercellular space (Mugford et al., 2016; Van Bel and Will, 2016; Mondal, 2020).

## Outlook

The rapidly growing field of effector studies, i.e., effectoromics, is uncovering the complexity of how insects modulate their hosts for their own benefit. It has become clear that herbivorous arthropods produce many proteins in their saliva, several of which influence the defense responses of their host plants. Optimal effectoromics research requires better genome assemblies and annotation resources, as these would facilitate the identification of duplicated multigene families, which might play important roles in the interactions of polyphagous insects such as *B. tabaci* with different host plants. Effectors are most often studied in plant–biotic interactions that involve specialized pathogens or pests, with the idea that effectors and their plant targets are in an evolutionary arms race. However, it is less clear how effectors of polyphagous insects evolve. To shed more light on this, it will be needed to generate genome-scale information of closely related specialists and generalists. So far, genome-wide comparisons have involved more divergent species (e.g., *M. persicae* and *A. pisum*). Whereas, these studies have provided information about large-scale evolutionary processes, such as chromosome organization, comparisons at this scale may be less useful for analyzing more recent evolutionary events involving effector genes. Hence, future research may focus on comparative genome analyses of closely related species with different plant host preferences. The *B. tabaci* species complex is a good candidate for this type of analysis, as there are many species with known host specificity (Malka et al., 2018). To functionally characterize candidate effectors gleaned from the comparative genome analyzes, further optimizations of whitefly RNAi and CRISPR approaches are required. Do these effectors truly contribute to insect feeding behavior, reproduction, and overall fitness? The answer to this question probably varies among plant species the insect may or may not colonize and whether the effector is more widely conserved or family/species specific within the hemipterans.

The plant interactors for some effector proteins were identified, providing more detailed insight into what these effectors accomplish in the plant cell. Altering the expression level of the corresponding plant genes leads to moderately altered levels of resistance. This incomplete or partial level of resistance phenotype indicates that we are dealing with a polygenic trait (Kliebenstein, 2014; Corwin and Kliebenstein, 2017; Du et al., 2020). The most likely explanation for this is that several proteins in the plant are targeted by effectors, that all have some impact on susceptibility. All of the data in hemipterans point in this direction. This information would have to be taken into account when breeding for resistance. This breeding objective could be met (i) via natural variation: as the genome sequences of host plants become more available, allelic variation in putative interaction sites could be detected *in silico*; and (ii) via EMS (Ethyl Methane Sulfonate) or CRISPR-based mutagenesis. Both approaches require a thorough understanding of the interaction domain of the plant protein and the effect of mutations in this domain on the phenotype of the plant. This would require complementation studies in which mutated forms of the plant protein are expressed in knock-out plants.

Other outstanding questions involve the localization of the effector proteins *in planta*. Although answering these questions will truly be challenging, several fundamental questions eventually need to be addressed to understand the functions of effectors *in planta*: In which cells are they active? Does this coincide with the cells in which the interactors are expressed? Are the effectors systemically transported? In order to better select effectors relevant to phloem-feeding insects, we need assays that are located in the phloem. The agroinfiltration assays described above have primarily involved the transformation of epidermal and mesophyll cells. One possible option is to adapt the phloem-localized GCaMP3 fluorescent protein-based [Ca^2+^]_cyt_ sensor, which reports increased [Ca^2+^]_cyt_ upon herbivory in *A. thaliana*, for use in the model plant of choice (Vincent et al., 2017; Toyota et al., 2018).

We hope that the field of insect-effector biology will grow in order to achieve the critical mass needed to study these topics in detail. Finally, the discovery that RNA molecules from insects, including sRNAs and long non-coding RNAs, are transported into plants has opened up a whole new field of research. However, the questions about these molecules also revolve around a central theme: What is their mode of action *in planta*, and how can we use this knowledge to increase plant resistance to whiteflies?

## Author Contributions

DN and RS conceived the manuscript. SD, PK, DN, and RS conceived the figure. SD made and further designed the figure. MM checked and updated the supplementary data. RS and SH critically revised the manuscript. PK extensively rewrote the manuscript. All authors contributed to writing and discussions on the content and read and approved the manuscript.

## Conflict of Interest

The authors declare that the research was conducted in the absence of any commercial or financial relationships that could be construed as a potential conflict of interest.
