# Supplementary material for: Spotlight on the Roles of Whitefly Effectors in Insect–Plant Interactions
Source: Front Plant Sci. 2021 Jul 2;12:661141. doi: 10.3389/fpls.2021.661141 (PMC8283192; doi:10.3389/fpls.2021.661141)
Supplement: Supplementary file 1 [file Table_1.DOCX]

**Supplemental table 1.** Effector molecules found in phloem-feeding insects other than *B. tabaci*.

| **Effector name** | **Mode of action** | **Phloem-feeding insect** | **References** |
| --- | --- | --- | --- |
| Putative elicitors between 3 and 10 kD in size | Induce resistance against aphid infestation in *A. thaliana* | *Myzus persicae* (aphids) | De Vos and Jander, 2009 |
|  |  |  |  |
| Mp42 | Reduces aphid fecundity in *N. benthamiana* | *Myzus persicae* (aphids) | Bos *et al*., 2010 |
|  |  |  |  |
| Mp2 (Plnt02) | Increases aphid fecundity in *N. benthamiana* and *A. thaliana* | *Myzus persicae* (aphids) | Pitino and Hogenhout, 2013 |
|  |  |  |  |
| Mp56  Mp57  Mp58 | Reduce aphid fecundity in *N. tabacum* and *A. thaliana* | *Myzus persicae* (aphids) | Elzinga *et al*., 2014 |
|  |  |  |  |
| Mp55 | Increases aphid fecundity in *N. tabacum, N. benthamiana*, and *A. thaliana*  Diminishes the accumulation of 4-methoxy-indol-3-ylmethyl glucosinolate, callose, and hydrogen peroxide upon aphid feeding in *A. thaliana*  Attracts aphids when expressed in *A. thaliana* | *Myzus persicae* (aphids) | Elzinga *et al*., 2014 |
|  |  |  |  |
| Mp10 | Localizes to the cytosol and chloroplasts of plant mesophyll cells adjacent to the aphid stylet track in *A. thaliana*  Suppresses the oxidative burst induced by flg22 *in N. benthamiana*  Reduces aphid fecundity in *N. benthamiana*  Induces chlorosis in *N. benthamiana* | *Myzus persicae* (aphids) | Bos *et al*., 2010  Mugford *et al*., 2016 |
|  |  |  |  |
| MpC002 | Localizes to the apoplastic space of mesophyll tissue around the stylet in *A. thaliana*  Increases aphid fecundity in *N. benthamiana*  Increases aphid fecundity in *N. benthamiana* and *A. thaliana* | *Myzus persicae* (aphids) | Mugford *et al.*, 2016  Bos *et al.*, 2010  Pitino and Hogenhout, 2013 |
|  |  |  |  |
| Mp1 (Plnt01) | Interacts with Vacuolar protein sorting associated protein 52 (VPS52) from *A. thaliana* and *S. tuberosum*  Reduces aphid fecundity in *N. benthamiana*  Aphids reduce the expression of VPS52 in *A. thaliana*  Increases aphid fecundity in *A. thaliana*  Localizes to the sheath surrounding the aphid stylet at feeding sites in *A. thaliana* | *Myzus persicae* (aphids) | Rodriguez *et al*., 2017  Pitino and Hogenhout, 2013  Mugford *et al.*, 2016 |
|  |  |  |  |
| Armet | Induces pathogen resistance by promoting SA accumulation in *N. benthamiana*  Activates signaling pathways associated with plant–pathogen interactions, mitogen-activated protein kinase in *N. benthamiana* and *M. truncatula*  Plant diet instead of artificial diet increases Armet transcript levels in *V. faba*  Knockdown by RNAi disturbs aphid feeding behavior, shortens aphid life span, and stimulates saliva secretion in *V. faba*  Localizes to watery saliva of aphids and the phloem sap upon aphid feeding in *V. faba* | *Myzus persicae* (aphids)  *Acyrthosiphon pisum* (aphids) | Cui *et al.*, 2019  Wang *et al.*, 2015 |
|  |  |  |  |
| Putative effectors | Comparative analysis of salivary gene expression in different *A. pisum* biotype lines with clearly distinct host plant specificity. | *Acyrthosiphon pisum* (aphids) | Boulain *et al.*, 2019 |
|  |  |  |  |
| Me10  Me23 | Enhance aphid fecundity in *N. benthamiana* and *S. lycopersicum* (Me10) | *Macrosiphum euphorbiae* (aphids) | Atamian *et al.*, 2013 |
|  |  |  |  |
| Me47 | Is an active glutathione-S-transferase  Enhances *Myzus persicae* performance when expressed in *N. benthamiana*  Enhances *Macrosiphum euphorbiae* performance when expressed in *S. lycopersicum* but not *A. thaliana* | *Macrosiphum euphorbiae* (aphids) | Kettles and Kaloshian, 2016 |
|  |  |  |  |
| RpC002  Rp1 | Promotes host susceptibility by suppressing defense gene expression in *H. vulgare* | *Rhopalosiphum padi* (aphids) | Escudero-Martinez *et al.*, 2020 |
|  |  |  |  |
| NlSEF1 | Knockdown in nymphs elicits higher levels of Ca^2+^ and H_2_O_2_ but not JA, JA-Ile or SA in *O. sativa* and increases mortality and diminishes the nymph’s feeding capacity | *Nilaparvata lugens* (planthopper) | Ye *et al*., 2017 |
|  |  |  |  |
| NlEG1 | Is an active endo-β-1,4-glucanase  Allows the stylet to reach the phloem by degrading cellulose in plant cell walls  Knockdown reduces food intake, mass, survival, and fecundity on *O.* sativa | *Nilaparvata lugens* (planthopper) | Ji e*t al*., 2017 |
|  |  |  |  |
| Nl12  Nl16  Nl28  Nl43 | Induce HR in *N. benthamiana* and *O. sativa*  Enhance defense-related gene expression when expressed in *N. benthamiana* | *Nilaparvata lugens* (planthopper) | Rao e*t al*., 2019 |
|  |  |  |  |
| NlMLP | Mucin-like protein  Induces HR in *N. benthamiana* and *O. sativa* and the expression of defense-related genes and callose deposition in *N. benthamiana*  Knockdown disturbs salivary sheath formation and consequently insect performance on *O. sativa* | *Nilaparvata lugens* (planthopper) | Shangguan *et al*., 2018 |
|  |  |  |  |
| Nl40 | Induces chlorosis in *N. benthamiana*  Enhances defense-related gene expression when expressed in *N. benthamiana* | *Nilaparvata lugens* (planthopper) | Rao *et al*., 2019 |
|  |  |  |  |
| Nl32 | Induces a dwarf phenotype in *N. benthamiana*  Enhances defense-related gene expression when expressed in *N. benthamiana* | *Nilaparvata lugens* (planthopper) | Rao *et al*., 2019 |
|  |  |  |  |
| NlSP1 | Induces HR, H_2_O_2_ accumulation, expression of defense-related genes, and callose deposition in *N. benthamiana* and *O. sativa* (protoplasts)  Reducing *NlSP1* expression in *N. lugens* (via RNAi) decreases survival | *Nilaparvata lugens* (planthopper) | Huang et al., 2020 |
|  |  |  |  |
| Salivary DNaseII | DNA-degrading activity  Decreases H_2_O_2_ accumulation and callose deposition in *O. sativa*  Knockdown reduces fecundity, survival, and honeydew production on *O. sativa* | *Laodelphax striatellus* (planthopper) | Huang *et al*., 2019 |
|  |  |  |  |
| Mucin-like protein | Is highly expressed in the salivary gland and throughout all life stages of *S. furcifera* | *Sogatella furcifera* (planthopper) | Miao *et al.*, 2018 |
|  |  |  |  |
| List of effector candidate proteins | Present in watery saliva, secreted in artificial diet | *Nephotettix cincticeps* (planthopper) | Hattori *et al*., 2015 |
|  |  |  |  |
| NcSP75 | Knockdown reduces survival, life span, and reproduction and causes developmental retardation on *O. sativa* | *Nephotettix cincticeps* (planthopper) | Matsumoto and Hattori, 2018 |
|  |  |  |  |
| List of effector candidate proteins | Present in saliva, secreted in artificial diet | *Diaphorina citri* (psyllid) | Yu and Killiny, 2018 |

Atamian, H.S., Chaudhary, R., Cin, V.D., Bao, E., Girke, T., Kaloshian, I., 2013. In planta expression or delivery of potato aphid *Macrosiphum euphorbiae* effectors *Me10* and *Me23* enhances aphid fecundity. MPMI 26, 67–74. https://doi.org/10.1094/MPMI-06-12-0144-FI

Bos, J.I.B., Prince, D., Pitino, M., Maffei, M.E., Win, J., Hogenhout, S.A., 2010. A functional genomics approach identifies candidate effectors from the aphid species *Myzus persicae* (Green Peach Aphid). PLoS Genet 6, e1001216. https://doi.org/10.1371/journal.pgen.1001216

Boulain, H., Legeai, F., Jaquiéry, J., Guy, E., Morlière, S., Simon, J.-C., Sugio, A., 2019. Differential expression of candidate salivary effector genes in pea aphid biotypes with distinct host plant specificity. Front. Plant Sci. 10, 1301. https://doi.org/10.3389/fpls.2019.01301

Cui, N., Lu, H., Wang, T., Zhang, W., Kang, L., Cui, F., 2019. Armet, an aphid effector protein, induces pathogen resistance in plants by promoting the accumulation of salicylic acid. Phil. Trans. R. Soc. B 374, 20180314. https://doi.org/10.1098/rstb.2018.0314

De Vos, M., Jander, G., 2009. *Myzus persicae* (green peach aphid) salivary components induce defence responses in *Arabidopsis thaliana*. Plant, Cell & Environment 32, 1548–1560. https://doi.org/10.1111/j.1365-3040.2009.02019.x

Elzinga, D.A., De Vos, M., Jander, G., 2014. Suppression of plant defenses by a *Myzus persicae* (Green Peach Aphid) salivary effector protein. MPMI 27, 747–756. https://doi.org/10.1094/MPMI-01-14-0018-R

Escudero-Martinez, C., Rodriguez, P.A., Liu, S., Santos, P.A., Stephens, J., Bos, J.I.B., 2020. An aphid effector promotes barley susceptibility through suppression of defence gene expression. Journal of Experimental Botany 71, 2796–2807. https://doi.org/10.1093/jxb/eraa043

Hattori, M., Komatsu, S., Noda, H., Matsumoto, Y., 2015. Proteome analysis of watery saliva secreted by green rice leafhopper, *Nephotettix cincticeps*. PLOS ONE 10, e0123671. https://doi.org/10.1371/journal.pone.0123671

Huang, H.-J., Cui, J.-R., Xia, X., Chen, J., Ye, Y.-X., Zhang, C.-X., Hong, X.-Y., 2019. Salivary DNase II from *Laodelphax striatellus* acts as an effector that suppresses plant defence. New Phytologist 224, 860–874. https://doi.org/10.1111/nph.15792

Huang, J., Zhang, N., Shan, J., Peng, Y., Guo, J., Zhou, C., Shi, S., Zheng, X., Wu, D., Guan, W., Yang, K., Du, B., Zhu, L., Yuan, L., He, G., Chen, R., 2020. Salivary protein 1 of brown planthopper is required for survival and induces immunity response in plants. Front. Plant Sci. 11. https://doi.org/10.3389/fpls.2020.571280

Ji, R., Ye, W., Chen, H., Zeng, J., Li, H., Yu, H., Li, J., Lou, Y., 2017. A Salivary endo-β-1,4-glucanase acts as an effector that enables the brown planthopper to feed on rice. Plant Physiol. 173, 1920–1932. https://doi.org/10.1104/pp.16.01493

Kettles, G.J., Kaloshian, I., 2016. The potato aphid salivary effector Me47 Is a glutathione-s-transferase involved in modifying plant responses to aphid infestation. Front. Plant Sci. 7. https://doi.org/10.3389/fpls.2016.01142

Matsumoto, Y., Hattori, M., 2018. The green rice leafhopper, *Nephotettix cincticeps* (Hemiptera: Cicadellidae), salivary protein NcSP75 is a key effector for successful phloem ingestion. PLOS ONE 13, e0202492. https://doi.org/10.1371/journal.pone.0202492

Miao, Y.T., Deng, Y., Jia, H.K., Liu, Y.D., Hou, M.L., 2018. Proteomic analysis of watery saliva secreted by white-backed planthopper, *Sogatella furcifera*. PLOS ONE 13, e0193831. https://doi.org/10.1371/journal.pone.0193831

Mugford, S.T., Barclay, E., Drurey, C., Findlay, K.C., Hogenhout, S.A., 2016. An immuno-suppressive aphid saliva protein is delivered into the cytosol of plant mesophyll cells during feeding 29, 8.

Pitino, M., Hogenhout, S.A., 2013. Aphid protein effectors promote aphid colonization in a plant species-specific manner. MPMI 26, 130–139. https://doi.org/10.1094/MPMI-07-12-0172-FI

Rao, W., Zheng, X., Liu, B., Guo, Q., Guo, J., Wu, Y., Shangguan, X., Wang, H., Wu, D., Wang, Z., Hu, L., Xu, C., Jiang, W., Huang, J., Shi, S., He, G., 2019. Secretome analysis and *in Planta* expression of salivary proteins identify candidate effectors from the brown planthopper *Nilaparvata lugens.* MPMI Vol. 32, No. 2, 2019, pp. 227–239. https://doi.org/10.1094/MPMI-05-18-0122-R

Rodriguez, P.A., Escudero-Martinez, C., Bos, J.I.B., 2017. An Aphid Effector Targets Trafficking Protein VPS52 in a Host-Specific Manner to Promote Virulence1[OPEN] 173, 12. https://doi.org/DOI: https://doi.org/10.1104/pp.16.01458

Shangguan, X., Zhang, J., Liu, B., Zhao, Y., Wang, H., Wang, Z., Guo, J., Rao, W., Jing, S., Guan, W., Ma, Y., Wu, Y., Hu, L., Chen, R., Du, B., Zhu, L., Yu, D., and He, G. 2018. A mucin-like protein of planthopper is required for feeding and induces immunity response in plants. Plant Physiol. 176:552-565. https://doi.org/DOI: https://doi.org/10.1104/pp.17.00755

Wang, W., Dai, H., Zhang, Y., Chandrasekar, R., Luo, L., Hiromasa, Y., Sheng, C., Peng, G., Chen, S., Tomich, J.M., Reese, J., Edwards, O., Kang, L., Reeck, G., Cui, F., 2015. Armet is an effector protein mediating aphid‐plant interactions. FASEB j. 29, 2032–2045. https://doi.org/10.1096/fj.14-266023

Ye, W., Yu, H., Jian, Y., Zeng, J., Ji, R., Chen, H., Lou, Y., 2017. A salivary EF-hand calcium-binding protein of the brown planthopper *Nilaparvata lugens* functions as an effector for defense responses in rice. Scientific Reports 7, 40498. https://doi.org/10.1038/srep40498

Yu, X., Killiny, N., 2018. The secreted salivary proteome of Asian citrus psyllid *Diaphorina citri*. Physiological Entomology 43, 324–333. https://doi.org/10.1111/phen.12263
